# Rapid Torque Production of the Knee Extensors: An Integrative Analysis of Neuromuscular and Muscle–Tendon Determinants

**DOI:** 10.1111/sms.70328

**Published:** 2026-07-02

**Authors:** Francesco Salvaggio, Baptiste Bizet, Samuel D′Emanuele, Michele Trinchi, Tommaso Berbenni, Alberto Rainoldi, Federico Schena, Cantor Tarperi, Andrea Monte, Gennaro Boccia

**Affiliations:** ^1^ Neuromuscular Function Research Group, School of Exercise and Sport Science University of Turin Turin Italy; ^2^ Department of Medical Sciences University of Turin Turin Italy; ^3^ Department of Neuroscience, Biomedicine and Movement Sciences University of Verona Verona Italy; ^4^ Department of Clinical and Biological Sciences University of Turin Turin Italy

**Keywords:** explosive strength, HD‐sEMG, impulse, neuromechanics, rapid force production, rate of force development, tendon stiffness, ultrasonography

## Abstract

The physiological determinants of explosive strength are usually investigated separately. We aimed to quantify the relative contributions of muscle excitation, structural, and mechanical determinants of rapid torque production in the knee extensors in a multi‐domain framework. Twenty‐four young adults performed ballistic isometric knee extensions. High‐density surface electromyographic (HD‐sEMG) signals were recorded from vasti lateralis and medialis muscles. Patellar tendon stiffness and muscle architecture were assessed via ultrasound. Impulse and RTD_peak_ were outcomes. The predictors were from four domains: muscle excitation, muscle thickness, fascicle shortening velocity, and tendon stiffness. Linear regressions and LMG were used to quantify the weight of each predictor. The models explained substantial variance in impulse: R^2^ = 0.62 (0–50 ms), 0.76 (0–75 ms), 0.74 (0–100 ms), 0.61 (0–125 ms), 0.70 (0–150 ms). Early impulse (50 and 75 ms) was mainly explained by tendon stiffness and muscle excitation; mid‐phase impulse (100 ms) by fascicle velocity and tendon stiffness; late impulse (125 and 150 ms) by fascicle shortening velocity and muscle thickness. RTD_peak_ was explained (R^2^ = 0.70) by patellar tendon stiffness (~32% of impulse variance), fascicle velocity (~18%), muscle thickness (~12%), and muscle excitation (~8%). Four predictors across different neuromechanical domains explained up to 76% of the variance in rapid torque production. Interestingly, the determinants shift over time: tendon stiffness and muscle excitation dominate earlier, while fascicle dynamics and muscle size become more important later. RTD_peak_ was largely determined by tendon stiffness and a balanced contribution of muscular predictors.

## 1 Introduction

Explosive strength could be defined as the capability to produce high values of force/torque as quickly as possible [1, 2]. This torque expression capacity has been largely recognized as a key ability for tasks that involve rapid movements or sudden postural adjustments, such as those encountered in sport performance or during balance recovery [3, 4, 5], playing an important role during the entire lifespan.

The rate of torque development (RTD) is the most common measure of explosive strength, and it is computed as the increase in torque over time ($\frac{∆T}{∆t}$) from contraction onset, starting from a resting state [6] or as the maximal instantaneous rate of change in torque (RTD_peak_) [7]. However, the impulse (i.e., the integral of the torque‐time signal) may provide a more functional descriptor of mechanical output over short time windows (≤ 150 ms) [8], and may be a more precise measure than its RTD (time‐locked) counterpart since its bidimensional nature (i.e., area vs. linear distance) [9]. The RTD_peak_, on the other hand, has been shown to be the most reliable RTD measure and is widely used because it quantifies the maximal instantaneous capacity of the neuromuscular system to increase torque once activation has begun [10, 11], providing complementary information to that offered by impulse metrics. Despite the importance of explosive strength, its underpinning physiological mechanisms remain poorly understood [12].

Muscle contraction is a complex, multifaceted process that integrates neural/nervous factors with structural and mechanical parameters. During explosive contractions, the motor unit behavior plays a crucial role in determining the initial rise in torque. Indeed, motor units' firing rate [13] and doublet occurrence [14] have been shown to be strongly correlated with the initial rise in torque during explosive contractions. Later, the recruitment speed of motor units has been reported as slightly more important [15, 16]. However, because no structural/mechanical variables were collected alongside neural/nervous ones, previous studies have not quantified the relative weight of predictors in a more inclusive multi‐domain framework. In this regard, several mechanical parameters, such as tendon stiffness [17, 18, 19] and muscle belly gearing (i.e., the ratio between muscle shortening velocity and fascicle shortening velocity) [20], have been reported as positively correlated with RTD. Other structural parameters, such as pennation angle and muscle volume [21], or fascicle length and fascicle shortening velocity [22] contribute to the increase in torque rise during an explosive contraction, especially in later contraction phases (e.g., after 100 ms from torque onset). But again, the cited studies did not consider the neural counterpart, failing to address a broad and potentially complete set of determinants.

Prior attempts to evaluate the relative contribution of mechanisms underpinning explosive torque have investigated central (i.e., neural/nervous) and peripheral (i.e., musculotendinous) factors [23, 24, 25]. In this regard, the peripheral contributions have been investigated using muscle contractility, analyzing the torque response evoked by a single electrical stimulus (twitch) [12] or by brief high‐frequency stimulation trains (e.g., octets at 300 Hz) [26]. These indices are thought to reflect the combined influence of peripheral properties involved in torque production, including excitation–contraction coupling, muscle‐tendon stiffness, and fascicle behavior [24]. In contrast, maximal voluntary torque (MVT) represents the maximal torque‐generating capacity achieved under voluntary conditions and therefore integrates both central neural drive and peripheral muscle properties [27]. However, because both contractility measures and MVT are outputs of numerous underlying mechanisms, they provide limited insight into the specific mechanisms governing early impulse (or torque) production and RTD_peak_.

In summary, the current literature has investigated the physiological determinants of explosive strength mainly in isolation, a practice that likely overestimates the relative importance of individual factors. To address this knowledge fragmentation, we combined high‐density surface electromyography (HD‐sEMG) with ultrasonography of tendon and muscle behavior during explosive isometric contractions to quantify the relative contributions of neural, structural, and mechanical determinants of explosive strength. The knee extensor muscles were selected as the experimental model given their central role in daily activities such as walking and running, as well as in postural control [28, 29]. We hypothesize that the early phase of explosive contraction is primarily driven by neural factors, whereas the later phase is driven by mechanical and structural factors. Additionally, we expected that our multi‐domain model would explain a greater variance in explosive torque (R^2^) than previous studies that considered measures from a single or limited number of domains (e.g., only neural/nervous factors).

## 2 Materials and Methods

### 2.1 Participants

A total of 15 young females (26 ± 3 years old) and 17 young males (25 ± 3 years old) participated in this study. The participants were healthy and reported no neuromuscular injury or disease within the 6 months preceding the experiments. All participants provided written informed consent before the measurements began. The study was approved by the Ethical Advisory Committee of the University of Turin (approval number 0528472, September 16, 2024) in accordance with the Helsinki Declaration. Of the 32 volunteers, eight were excluded from the final analysis either because they dropped out or did not provide acceptable ultrasound data. A total of 24 participants were included in the results.

### 2.2 Experimental Protocol

Each volunteer participated in a single experimental session of about 60 min. Strenuous exercise and caffeine were avoided 24 h and 4 h before the experimental session, respectively. The experimental session involved a specific and progressive warm‐up comprising submaximal isometric knee extension contractions. Participants were asked to exert torque gradually to ~25% of their perceived maximal torque and to maintain this level for 3 s. Three repetitions were performed, and the same was repeated for ~50%, ~75%, and ~90% of perceived maximal torque. The force signal was displayed live for visual reference throughout the warm‐up using the manufacturer‐supplied software (OT BioLab + software version 1.6.0.0, OT Bioelettronica, Turin, Italy). After that, two incremental protocols, one with a series of single electrical stimuli and one with octets (eight stimuli at 300 Hz), were delivered to determine the minimum intensity required to elicit the maximal M‐wave and the peak stimulated torque and RTD, respectively. Two maximum voluntary fixed‐end contractions (MVC) were then recorded 2 min apart. After 2 min, familiarization with explosive contractions consisted of performing ballistic (burst‐like) pulses by reaching at least 70% of the MVT “as fast as possible” and relaxing instantly [30, 31]. The 70% target was displayed as a red line in the monitor. An experienced operator decided when to finish the familiarization based on the absence of countermovement and no further improvement in RTD. Finally, 15 explosive (ballistic) contractions were performed.

In short, the protocol performed by the participant consisted of: warm‐up; electrical evoked contraction; 2–3 MVCs; explosive contractions.

### 2.3 Participants Preparation

During the experimental procedure, participants sat on a custom‐made chair and were secured with a seatbelt around their hips and braces around their shoulders to prevent sliding during the test. The knees and hips were flexed at 85° and 100° (180° being full extension), respectively. For setup convenience, the right ankle was tightly strapped to the strain gauge load cell (546QD‐220 kg; DSEurope, Milan, Italy) about 2 cm above the malleolus. To avoid pain and maintain stiffness, a hard shin protector was placed between the tibia and the thrust surface of the load cell, with the latter being perpendicular to the tibial alignment. The force signal was displayed live for visual reference throughout the session. To convert the force signal measured by the load cell into torque, the external moment arm was collated using a tape measure as the distance from the knee joint center of rotation (lateral femoral condyle) to the center of the load cell.

Before data collection, skin preparation for HD‐sEMG recording consisted of shaving the thigh skin, gently abrading it with an abrasive paste, cleaning it with water, and then drying it. One HD‐sEMG grid of 64 electrodes (13 rows × 5 columns, 8 mm inter‐electrode distance, gold‐coated; GR08MM1305, OT Bioelettronica, Turin, Italy) was placed on each muscle, vastus lateralis (VL) and vastus medialis (VM), using a disposable bi‐adhesive foam layer with holes adapted to the electrode grids (SpesMedica, Battipaglia, Italy). Grids were aligned with the supposed fiber orientation and placed distally to leave space for the ultrasound probe. The electrode cavities of the matrices were filled with 20–30 mL of conductive paste (Spes‐Medica, Battipaglia, Italy). The electrode arrays were fixed with an extensible tape (Hypafix, Smith and Nephew, Melbourne, Australia). The reference electrode (24 mm, model: CDE‐S, OT Bioelettronica, Turin, Italy) was placed on the medial femoral condyle of the same limb, and a wet strap ground electrode was placed around the left ankle.

An ultrasound apparatus with a 6‐cm linear‐array probe operating at 90 Hz (Telemed Mycrus Ext‐1, Lithuania) recorded patellar tendon displacement during MVCs. The ultrasound device was synchronized with the analog‐digital converter, and therefore with the force and HD‐sEMG amplifier, employing a 3 V external trigger. During the ballistic contractions, the ultrasound probe was placed over the VL muscle belly to assess fascicle length and aponeuroses changes. The sample rate was increased to 110 Hz, and the probe was attached to the skin approximately at 50% of the femoral length, aligned on the muscle belly, and corrected for the superficial and deep aponeurosis to have a clear image of the perimysial connective intramuscular tissue (i.e., indicative of the muscle fascicle structure). An experienced operator manually held the probe and asked to repeat a contraction if the recording was flawed.

### 2.4 Data Collection

**FIGURE 1:**
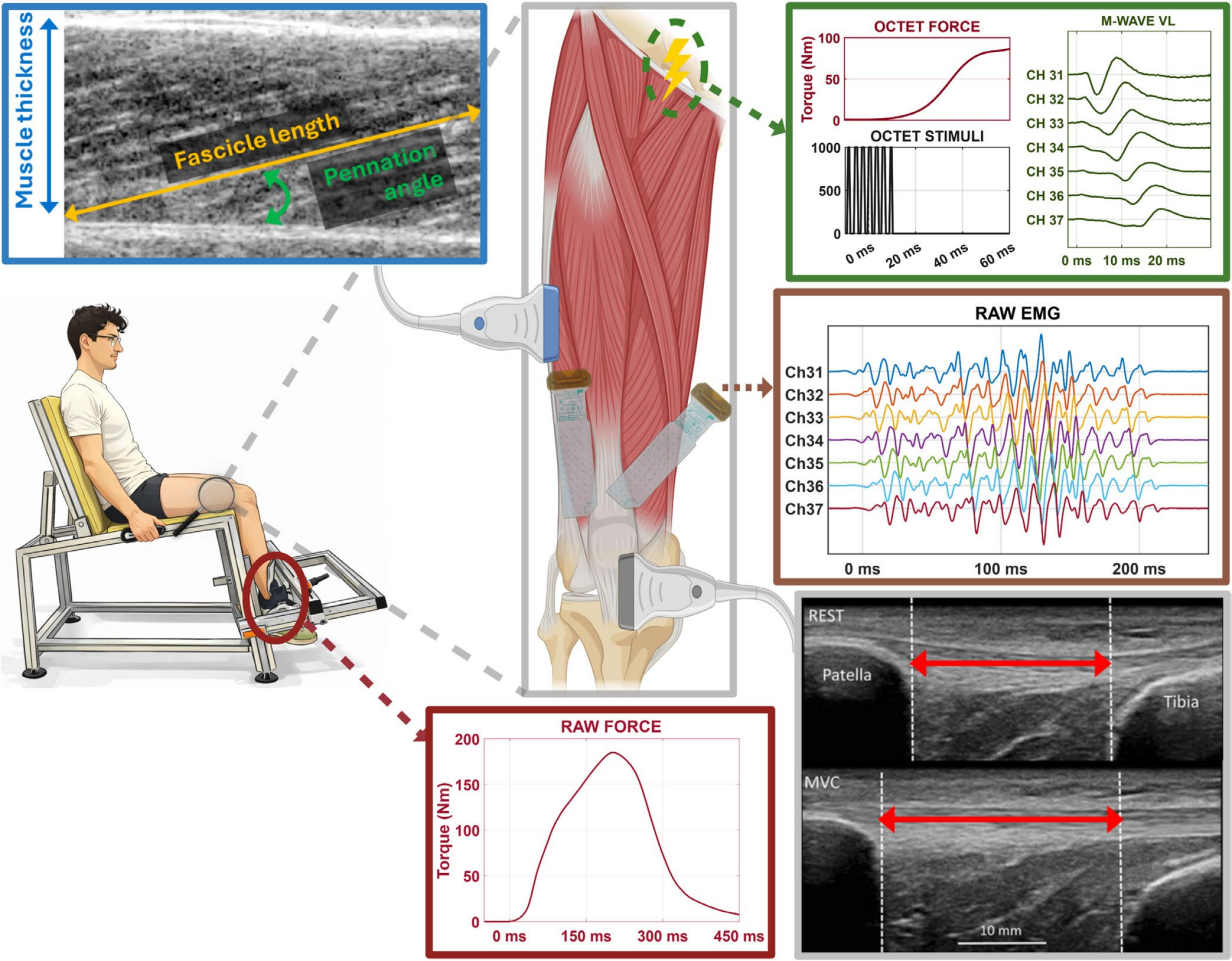
Representation of the experimental setup and the data acquired. The signals visualized in the figure are shown for illustrative purposes and may not match across plots, nor were they acquired together (e.g., within a measurement session, evoked signals were recorded prior to the voluntary ones).

An illustration of all data acquired during the measurements is presented in Figure 1. Torque and HD‐sEMG were acquired throughout the session, amplified with a gain of 500 and 150, respectively, sampled at 2048 Hz, and converted to digital data using a 16‐bit A/D converter (Quattrocento; OT Bioelettronica, Turin, Italy). HD‐sEMG was recorded in a monopolar configuration.

#### 2.4.1 Evoked Contraction

A constant‐current, variable‐voltage stimulator (Digitimer DS7A, Hertfordshire, UK) delivered a 0.2 ms square‐wave pulse with a maximum voltage of 400 V. The cathode (2 cm diameter, Meditrace 100 Kendall; Tyco, Markham, ON, Canada) and the anode (5 × 10 cm; Compex, Ecublens, Switzerland) were placed over the femoral nerve at the level of the femoral triangle below the inguinal ligament and just below the great trochanter, respectively. During the stimulation, the experimenter pressed the cathode with their thumb to reduce the electrode‐nerve distance and enhance the response to the stimulus [32]. Single electrical stimuli were delivered starting with 20 mA intensity and increasing in 20 mA increments, with a 30‐s rest between pulses. The same procedure was then repeated using octets. Via the manufacturer‐supplied software, the M‐wave was monitored and recorded from a representative EMG channel (visually inspected) of the vastus lateralis (VL), whereas torque and RTD (extracted from the torque signal) were inspected within a 300‐ms window triggered by the pulse generator. The stimulation intensity corresponding to the maximal response (M‐wave or RTD) was identified and subsequently increased by 20% to ensure supramaximal stimulation [26]. The median stimulation intensity used was 120 mA. Two single‐pulse and two octet stimuli were delivered, separated by 15 s, and used for later analysis.

#### 2.4.2 Voluntary Contractions

After the evoked contractions, a familiarization with the explosive ballistic voluntary contractions was performed, as suggested by Maffiuletti et al. [9]. Participants were instructed to “push as fast as possible”. To prevent ballistic contractions from resulting in only small torque outputs (albeit highly explosive), participants were required to reach at least 70% of their highest torque signposted by a red line on the live feedback display [31]. When a trial fell below this threshold, participants were encouraged to push slightly harder while maintaining the primary emphasis on producing torque as rapidly and explosively as possible, without aiming for maximal torque [33]. An experienced operator stopped the execution whenever no further improvement in the RTD was observed. Then, two MVCs, interspaced by a 2‐min rest, were performed to measure MVT and patellar tendon stiffness. An additional contraction was performed if the last attempt was 5% higher than the previous. Finally, 15 ballistic contractions spaced by 15 s rest were recorded. HD‐sEMG activity and ultrasound data were recorded during the entire burst‐like contractions protocol as reported above.

### 2.5 Data Analysis

#### 2.5.1 Torque Signals

The force signal was low‐pass filtered (200 Hz, 4th‐order Butterworth) and transformed into torque by multiplying it by the external moment arm. An investigator manually selected the contraction onset after visualizing the torque signal with a constant y‐axis scale of 1 N and with an x‐axis scale initially set to 500 ms [34]. Any countermovement visible in the torque signal resulted in discarding the contraction. After zooming to evaluate the baseline noise, the onset was selected as the last trough within the baseline noise envelope. For the best three contractions (based on torque at 75 ms), impulse metrics were computed as the integral of the torque‐time curve time‐locked to the first 50, 75, 100, 125, and 150 ms from torque onset (impulse50 to impulse150). The impulse metrics were then averaged across the three best contractions as suggested by Speedtsberg et al. [35]. The same computation was used for octet‐evoked contractions, although only the 0–50 ms window was kept since the octet is not sufficient to produce maximal explosive strength beyond that time window [26] and therefore longer windows would not represent maximal peripheral capacity for explosive torque production.

#### 2.5.2 HD‐sEMG Signals

HD‐sEMG signals were band‐pass filtered (50–500 Hz, 4th‐order Butterworth) and converted into single‐differential signals. Channels resulting from the differential of electrodes placed side by side were discarded, finally yielding 59 channels per grid. Channels with excessive noise or artifacts were removed through visual inspection. From the remaining channels, those that showed clear signal propagation were selected for further analysis. On average, six channels per column (range: 4–8) were included, resulting in approximately 30 EMG channels per muscle.

Onset detection was performed by first rectifying and then averaging all available EMG signals for each muscle (this procedure was applied only to onset detection, not to the signal processing phase). The EMG onset was selected on a 500‐ms window centered around the torque onset, where the EMG was plotted for each contraction. The onset was chosen as the last trough within the envelope of the baseline noise, as for the torque signal. The same procedure was used for evoked contractions, with particular attention to avoiding the stimulation artifact. From the same best three contractions selected from torque at 75 ms, the root mean square (RMS) of each single differential EMG signal was computed for all predefined time windows, considering VM onset for both muscles, as it was activated sooner (i.e., higher electromechanical delay). The RMS was averaged across all available channels for each muscle and normalized to the RMS of EMG computed from 500 ms around the MVT (peak torque during MVC) of the corresponding muscle. Finally, the normalized RMS averaged across VL and VM was retained to quantify overall quadriceps EMG activity in a single index [23, 24, 36]. The resulting variables are reported as EMG_vasti_50, EMG_vasti_75, EMG_vasti_100, EMG_vasti_125, and EMG_vasti_150.

Muscle fiber conduction velocity (MFCV) of VM and VL was estimated using an algorithm designed for multichannel EMG signals recorded during ballistic contractions [37]. The algorithm provides accurate estimates over epochs as short as 50 ms. Accordingly, MFCV was computed over the five time windows from the EMG onset of each muscle. The M‐wave MFCV was calculated from the same electrode columns used for the voluntary contractions, and relative values were obtained by normalizing to M‐wave velocity. MFCV estimates from voluntary and evoked contractions were averaged across the five electrode columns for each muscle [23].

#### 2.5.3 Ultrasound Data

Resting muscle architecture was analyzed using video recordings of MVCs. VL muscle thickness (MT), pennation angle (PA), and fascicle length (FL) at rest were obtained from two frames 2 s before the torque onset. The frames were manually analyzed by a single operator using Image J software (1.53a, Wayne Rasband National Institute of Health, USA) and averaged. Muscle thickness was defined as the distance between the deep and superficial aponeuroses, whereas pennation angle was defined as the angle between the collagenous tissue and the deep aponeurosis. FL was then manually calculated. A half‐cohort, including both sexes, was reanalyzed by the same operator to check the intra‐rater reliability. The ICC and CV were 0.98% and 3.4%, respectively.

During the MVC, patellar tendon displacement was evaluated by manually tracking the deep insertion of the tendon at the patella and tibial tuberosity using Tracker Motion (v 4.3). The torque‐elongation relationship was fitted with a second‐order polynomial fit forced through zero. Finally, patellar tendon stiffness was calculated as the slope of the torque‐patellar tendon elongation relationship over two intervals: 0%–50% and 50%–100% of MVC [38]. We selected these two torque intervals because they include the torque levels used in the first and last parts of an explosive short contraction, respectively.

During ballistic contractions, a validated semi‐automatic tracking algorithm (UltraTrack v. 5.5) was used to quantify fascicle and belly displacement frame by frame starting at torque onset. At the end of auto‐tracking, every frame of the tracked parameters was visually inspected to assess the algorithm's accuracy. Whenever the fascicle or belly length was deemed inaccurate, the two points defining the muscle fascicles were manually repositioned. Fascicle shortening velocity was calculated as the first derivative of the fascicle length–time curve. Muscle shortening velocity was computed as the product of fascicle velocity and the change in the fascicle pennation angle cosine [39]. Muscle belly gearing was computed as the ratio between belly and fascicle velocity. As in the case of impulse data, fascicle velocity, muscle velocity, and belly gearing were calculated over the five time windows from torque onset and averaged across the three best contractions based on the torque at 75 ms.

#### 2.5.4 Methods for Predictor Selection

As outlined in the introduction, the selected dependent variable is impulse (torque‐time integral), as it carries more information than the more conventional RTD [9], despite a very strong correlation between the two (*r* = 0.91–0.97). In previous studies, predictors were automatically included in the statistical analysis of determinants using stepwise linear regression. Although practical, this approach could exclude some mechanistically relevant domains and predictors just because another correlated predictor entered the regression first. This would have led to a harder interpretation of the results. Instead, our approach wants to select predictors from “domains” that represent distinct mechanisms. Given the sample size (*N* = 24) and to avoid overfitting, we restricted the number of predictors to four, each representing a distinct neuromechanical domain specified a priori: (a) muscle excitation; (b) muscle anatomical characteristics; (c) muscle behavior; (d) tendon mechanics.

A broad number of metrics, based on physiological rationale and evidence, was computed before being screened using a two‐step selection procedure (the most relevant are reported in Tables S1 and S2). When multiple variables conveyed overlapping information (e.g., fascicle vs. muscle‐belly velocities), within‐domain, we retained the metric that (i) exhibited the strongest bivariate association with impulse and (ii) showed the lowest redundancy with the other predictors, assessed by the correlation among predictors. After one representative predictor was retained from each domain, multicollinearity among the final predictors in the regression model was evaluated using variance inflation factors (VIF). The final predictor set was composed of four metrics:

1. **Muscle excitation**: the RMS of VL and VM EMG activity, normalized to the RMS during MVT, was included as an overall index of muscle excitation to the quadriceps [24, 27].
2. **Muscle anatomical characteristics**: VL muscle thickness was included as a surrogate of muscle volume, given its established contribution to maximal and explosive torque production [27].
3. **Muscle behavior**: VL fascicle shortening velocity was selected, as it showed the strongest association with impulse and was highly redundant with muscle‐belly velocity and architectural gearing, indicating that the latter variables would introduce collinearity without providing additional explanatory value
4. **Tendon** **mechanics**: the patellar tendon stiffness was included because of its key role in transmitting quadriceps torque to the tibia

Muscle excitation and VL fascicle shortening velocity were time‐matched to the corresponding impulse windows (e.g., 0–50 ms predictors for the 0–50 ms impulse model, etc.). Muscle excitation variables were computed from time windows starting at the EMG activity onset of VM, while torque variables and fascicle shortening velocity were extracted from windows starting at the torque onset. Muscle thickness was constant across models. Patellar tendon stiffness was torque‐matched to the level of torque attained within each window: stiffness measured from 0% to 50% MVT torque‐elongation curve was used for the impulse50 and impulse75 models, while stiffness computed from 50% to 100% MVT was used for the impulse100, impulse125, and impulse150 models, as torque at 100 ms was already higher than 50% of MVT.

As a secondary analysis, octet‐evoked RTD_50_ was entered into an additional model to examine whether contractile properties (i.e., when the central nervous system is bypassed using peripheral nerve stimulation) explained additional impulse variance beyond voluntary neuromechanical determinants. This variable was not considered for selection among the predictors as it is a complex interaction of more basic predictors [24].

### 2.6 Statistical Analysis

We performed progressive statistical analyses to identify the determinants of impulse across five time‐windows (0–50, 0–75, 0–100, 0–125, and 0–150 ms). As described in the previous paragraph, bivariate correlation analyses were performed between all investigated parameters and the time‐matched impulse to assess the directionality of their relationships for the variable selection step and to confirm results from previous studies (see correlation analysis in Supporting Information).

The primary analyses consisted of multiple linear regression models, one per time window, with the four predictors entered simultaneously to avoid order effects. The predictors were: average RMS of VL and VM normalized to their respective RMS during MVT, VL muscle thickness, patellar tendon stiffness, and VL fascicle velocity. For each model, we verified basic linear model assumptions using residuals versus fitted plots, Q‐Q plots, and influence diagnostics (leverage, Cook's D, and studentized residuals). Where mild heteroscedasticity appeared, we verified inferences using HC3 robust standard errors.

As a secondary analysis, the octet‐evoked RTD50 was entered into a separate model to test whether contractile properties explained additional variance in impulse beyond the single voluntary predictors.

We evaluated the importance of each predictor using dominance analysis, computed as the Lindeman–Merenda–Gold index (LMG). LMG is a statistical method that averages sequential R^2^ increases (ΔR^2^), thereby providing an average of the ΔR^2^ determined by each predictor across all possible orders of predictor entry in the regression [40]. The sum of the four LMG values finally equal to the model R^2^, allowing to rank the determinants by their importance.

## 3 Results

The descriptive data of the main parameters are reported in Table 1. The impulse increased as a function of time up to 150 ms of contraction onset. Patellar tendon stiffness calculated in the higher half was greater than that extracted from the lower half of the torque‐elongation curve. Interestingly, fascicle shortening velocity reached its peak at 100 ms and decreased slightly at 150 ms, whereas the vasti EMG was highest at 100 ms and remained constant up to 150 ms. Since RTD_peak_ occurred at 54 ± 13 ms (mean ± SD), two regression models were performed: one using predictors computed from 0 to 50 ms from their onsets (same used for the impulse50 model), and a second one using predictors from 0 to 75 ms from their onsets (as predictors in the impulse75 model). The model using predictors from 0 to 50 ms violated the linear regression assumptions, therefore the model with predictors from 0 to 75 ms was reported in the results. The latter model explained higher variance (R^2^≈0.70 vs. 0.61); a comparison between the two models is presented in Figure S1.

**TABLE 1:** Descriptive values of the main computed metrics.

Variable	Mean	SD	CoV
Impulse50 (Nm·s)	0.418	0.207	0.495
Impulse75 (Nm·s)	1.701	0.593	0.349
Impulse100 (Nm·s)	3.685	1.111	0.301
Impulse125 (Nm·s)	6.204	1.798	0.290
Impulse150 (Nm·s)	9.091	2.619	0.288
VL Th_m_ (cm)	2.421	0.482	0.199
k_pt_0‐50% MVT (Nm·mm^−1^)	1587.3	323.6	0.204
k_pt_50‐100% MVT (Nm·mm^−1^)	2027.3	180.9	0.089
VL V_f_50 (cm·s^−1^)	5.635	1.103	0.196
VL V_f_75 (cm·s^−1^)	8.491	0.668	0.079
VL V_f_100 (cm·s^−1^)	10.328	0.648	0.063
VL V_f_125 (cm·s^−1^)	8.650	0.579	0.067
VL V_f_150 (cm·s^−1^)	8.156	0.589	0.072
EMG_vasti_50	0.555	0.229	0.413
EMG_vasti_75	0.771	0.247	0.320
EMG_vasti_100	0.837	0.207	0.247
EMG_vasti_125	0.839	0.182	0.217
EMG_vasti_150	0.836	0.162	0.193
MVT (Nm)	171.2	53.6	0.313
RTD_peak_ (Nm·s^−1^)	1857.6	636.9	0.343
Evoked RTD50 (Nm·s^−1^)	1225.3	311.9	0.255
Evoked RTD_peak_ (Nm·s^−1^)	2720.2	680.9	0.250

*Note:* Descriptive values of the main metrics are reported as mean, standard deviation (SD), and coefficient of variation (CoV).

Abbreviations: EMG_vasti_50‐150, RMS from explosive contractions normalized to RMS at MVT averaged across VL and VM; Impulse50‐150, impulse from contraction onset to 50–150 ms; k_PT_, patellar tendon stiffness; k_PT_50MVC/k_PT_100MVC, Patellar tendon stiffness computed in the 0%–50% and 50%–100% MVT interval of the torque‐elongation curve; MVT, maximal voluntary torque; MVT, Maximal voluntary torque; octet‐evoked RTD50/octet‐evoked RTD_peak_, Octet‐evoked rate of torque development at 50 ms and peak; RTD_peak_, the peak rate of torque development; Th_m_, Muscle thickness; Vf50‐150, Fascicle shortening velocity, time‐matched to 0–50/100/150 ms windows; VL, vastus lateralis; VM, vastus medialis.

### 3.1 Correlations and Linear Regressions

The pairwise correlation coefficients between the dependent variables (i.e., impulses or RTD_peak_) and all predictors, including selected and unselected ones, are reported in the Supporting Information, section on correlation analysis. As expected, complex contractility metrics like MVT and octet‐evoked RTD presented higher correlation with later impulse time windows, with an r of 0.68 and 0.65 at 0–100 ms, 0.93 and 0.78 at 0–125 ms, and 0.85 and 0.81 at 0–150 ms, respectively, while 0.64 and 0.69 with RTD_peak_. Surprisingly, not only EMG_vasti_, but also k_pt_ were the predictors that best correlated with early impulse, with *r* = 0.66 and 0.60, respectively, at 0–50 ms, and 0.64 and 0.77, respectively, at 0–75 ms.

From multiple linear regressions, standardized beta coefficients and their significance, plus overall models' coefficients of determination (R^2^) are reported in Table S3. All models were fitted with ordinary least squares (OLS). The regression models for impulses 50, 75, 100, 125, and 150 explained 0.62, 0.76, 0.74, 0.61, and 0.70 of the variance, respectively, while the RTD_peak_ model with predictors from 0 to 75 ms yielded an R^2^ of 0.70. The octet‐evoked RTD_50_ was omitted by further discussion because no significant R^2^ increases were detected after adding it as an additional predictor to the impulse50 and RTD_peak_ regression models.

The coefficients resulting from the commonality analysis, which decompose each model R^2^ into unique and shared variance components for the selected predictors, are reported in the Table S4.

### 3.2 Dominance Analysis

**FIGURE 2:**
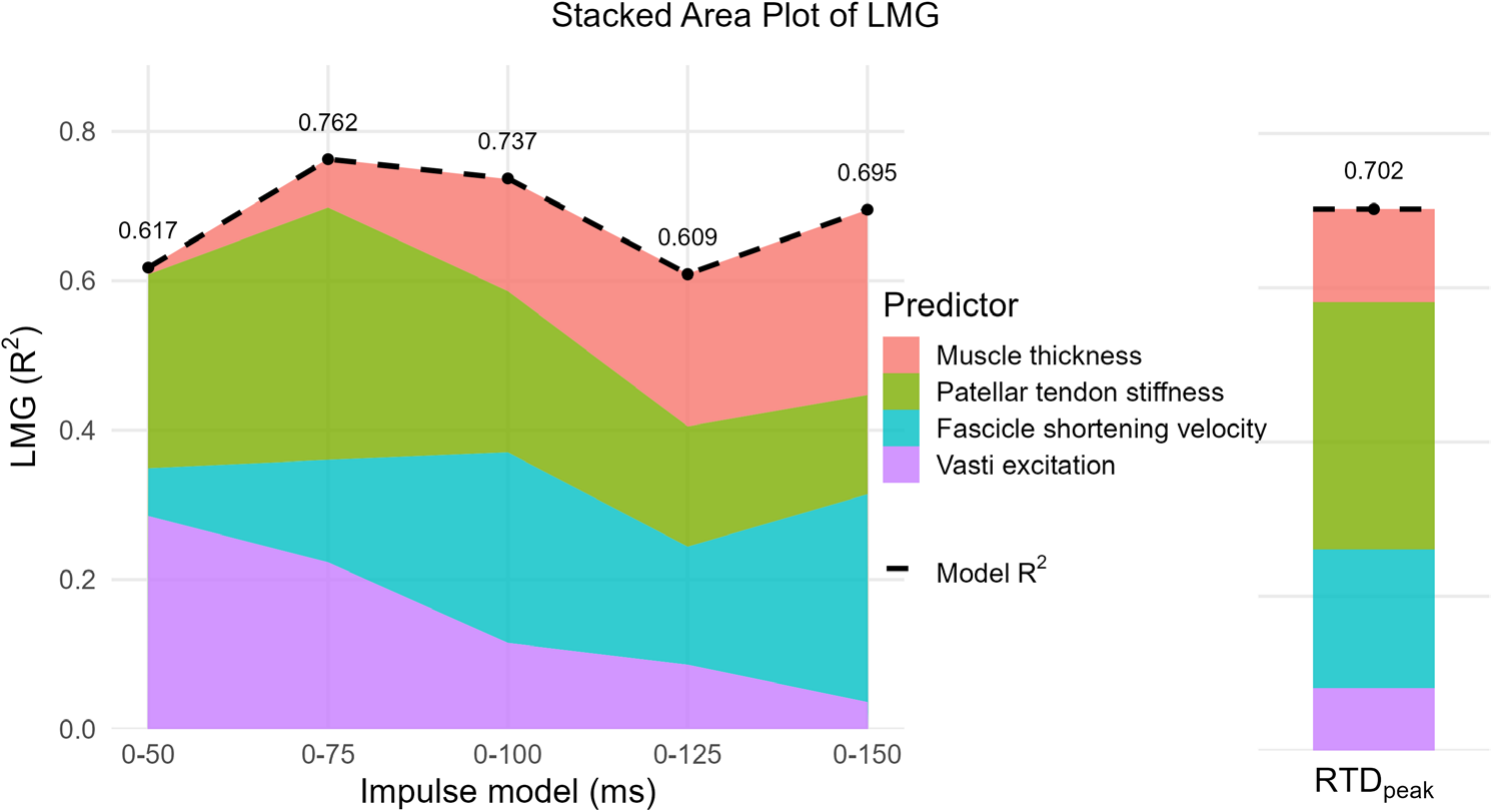
Stacked areas visualization of the LMG scores (R^2^) accounted by each determinant in consecutive impulse time windows and at RTD_peak_. Muscle thickness computed as the distance between superficial and deep aponeuroses of VL muscle 2 s before the contraction; Patellar tendon stiffness either computed from 0% to 50% and 50% to 100% MVT interval of the torque‐elongation curve, stiffness from 50% to 100% MVT was entered in impulse100 to 150 regression models, while that computed from 0% to 50% MVT was used for the earlier windows; Fascicle shortening velocity of VL was time‐matched to impulse; Vasti excitation is computed as the root mean square (RMS) of VL and VM normalized to RMS of MVC and averaged across VL & VM; RMS windows are time matched to impulse but start from EMG onset of VM.

For the dominance analysis, LMG scores are visualized as a stacked area plot over consecutive time windows for impulse, and as a stacked bar plot for RTD_peak_ (Figure 2). For a plain text report of LMG scores see Table S5.

Across all conditions, the models explained a substantial proportion of the variance (R^2^ = 0.609–0.762). For the 50‐ms impulse window, muscle excitation showed the largest contribution (LMG = 0.285), followed by k_pt_ (0.259), V_f_ (0.064), and Th_m_ (0.009). At 75 ms, the k_pt_ had the greatest weight (0.338), followed by muscle excitation (0.223), V_f_ (0.137) and Th_m_ (0.065). For impulse100, the largest contributions were observed for V_f_ (0.255) and k_pt_ (0.216), followed by Th_m_ (0.15) and EMG (0.115). Impulse from 0 to 125 ms was explained by Th_m_ (0.204), followed by k_pt_ (0.161), V_f_ (0.158) and EMG_vasti_ (0.086). Finally, for impulse150, the largest contributions were observed for V_f_ (0.278) and Th_m_ (0.248), followed by k_pt_ (0.133) and muscle excitation (0.036).

For RTD_peak_, k_pt_ had the highest contribution (LMG = 0.32), followed by V_f_ (0.18), Th_m_ (0.121), and EMG (0.081).

## 4 Discussion

This study combined HD‐sEMG and ultrasound data to quantify the relative contribution of different neuromechanical mechanisms underlying the explosive strength of the knee extensors. In agreement with our hypotheses, our multi‐domain model explained greater explosive strength variance (impulse or RTD R^2^) than previous studies that analyzed only one domain (not considering complex metrics like MVT, octet‐evoked RTD, etc. as predictors), such as Del Vecchio et al. [15] explaining R^2^ 0.45–55 from motor unit behavior, Van Hooren et al. [20] reporting r^2^ 0.38–0.58 from muscle‐tendon characteristic, and D'Emanuele et al. [23] finding r^2^ 0.39 between muscle excitation and RTD.

On the other hand, contrary to our hypotheses, the impulse at 50 ms was primarily explained by an interplay between patellar tendon stiffness and muscle excitation, rather than by neural/nervous parameters alone. At 100 ms, patellar tendon stiffness remained a key contributor, whereas VL fascicle shortening velocity emerged as the dominant determinant, showing a negative association with impulse (i.e., for a given contraction, the lower the shortening velocity, the higher the torque‐velocity potential, which in turn increases the torque rise). In the late phase of explosive torque production, VL fascicle shortening velocity was the main determinant, with VL muscle thickness and patellar tendon stiffness providing secondary contributions. For RTD_peak_, patellar tendon stiffness was the main determinant, whereas fascicle shortening velocity played a supportive role followed by muscle thickness, and with a modest contribution of muscle excitation.

### 4.1 Confirmation of Previous Results

In the first step, bivariate correlation analyses were performed between each neuromechanical predictor and each time‐matched impulse value (Table S1) to examine pairwise associations. In addition to the initial predictor selection, this step was used to confirm consistency between our and previous studies regarding the most commonly used metrics [23, 24]. As already mentioned, different mechanisms appear to underlie the early and late phases of rapid torque production, with their relative contribution likely changing throughout the rising portion of the torque‐time curve [41]. Our correlation analysis (Table S1) remarks that early impulse/RTD has a weak association with MVT [12] and is primarily correlated with muscle excitation [41]. Conversely, late impulse/RTD was strongly correlated with MVT and with contractile properties, that is, octet‐evoked torque/RTD, in line with previous studies [12, 24, 42].

### 4.2 Methodological Choices

We aimed to predict impulse generation using four analytical predictors that together explained up to 76% of the variance: (1) vastus lateralis muscle thickness, (2) muscle excitation of the vasti, (3) fascicle shortening velocity, and (4) patellar tendon stiffness. As shown later, our multi‐domain model assigns lower weights to each variable than previous models that examined each domain in isolation (i.e., nervous/neural vs. muscle‐tendon behavior vs. muscle morphology). This discrepancy does not imply a contradiction; rather, it is expected because single‐domain or bivariate models inevitably attribute to one predictor the variance that is actually shared with other correlated variables (i.e., multicollinearity). For instance, when only neural factors are analyzed as determinants of explosive torque, they may appear to contribute more strongly simply because they capture variance that is also explained by other physiological, architectural, or mechanical determinants. Our goal, therefore, was to assign a more appropriate weight to each domain to overcome the current fragmentation of knowledge regarding rapid torque production. To this end, we employed LMG (dominance analysis) on top of multiple linear regressions, to quantify the weight of each determinant of explosive torque production by correctly redistributing the overlapping variance of the included predictors [40].

Among metrics discarded in the linear regression, the octet‐evoked RTD represents a torque output measure that depends partly on muscle size and muscle‐tendon unit stiffness [17, 19], which are included as individual variables in the present study, and partly on fiber type composition [43], which we did not measure. In any case, including octet‐evoked RTD did not improve R^2^ in our regression models, suggesting that the set of predictors selected here was sufficient to capture the contractile response to octet stimulation.

### 4.3 Neural/Nervous Contributions

Previous studies of RTD determinants, which considered contractile measures or EMG‐derived metrics, have shown the key role of muscle excitation in generating early explosive torque. In our study, muscle excitation explained ~28.5% of the impulse variance at 50 ms, ~22.3% at 75 ms, ~11.5% at 100 ms, ~8.6% at 125 ms, and ~8.1% at 150 ms. Previously, Folland et al. [24] reported that quadriceps excitation contributed to ~37% of the variance in RTD at 0–50 ms, with no significant role at 0–100 ms or 0–150 ms. Similarly, D'Emanuele et al. [23] confirmed a significant 36% of R^2^ in torque at 50 ms explained by normalized RMS (computed in the same fashion as in this study), while no significant contribution from EMG appeared at 100 and 150 ms. Magrini et al. [44] applied linear regression and found that quadriceps excitation, although measured from maximal contractions, is a significant predictor of RTD 0–50 ms. More specifically, Del Vecchio et al. [15] found that motor unit recruitment speed and discharge rate explained 45% and 55% of the variance in impulse at 50 ms, respectively. These results were later confirmed in a computational study by Dideriksen et al. [16]. Compared to the motor unit metrics from the study by Del Vecchio et al. [15], our results report a smaller amount of variance explained by muscle excitation. This might be due to (1) the fact that the RMS of EMG signals is not a direct measure of motor‐unit activity, and is affected by amplitude cancellation due to the overlapping of positive and negative phases of individual action potentials [45]; (2) different muscles being measured, that is, tibialis anterior vs. knee extensors, and (3) the weight of the predictors is fairly reassigned in the present study (i.e., less overestimation from shared variance). The latter option is valuable because the variance shared by EMG_vasti_ and the impulse at 0–50 ms is 0.43 R^2^ (squared correlation coefficient; see Table S1), which is much larger than the final 0.28 R^2^ resulting from the LMG computation. This difference between bivariate and multivariate results underscores that a substantial part of the variance that impulse shared with EMG was also shared with other predictors and was thus redistributed using LMG computation.

Of note, MFCV has been suggested to provide an indirect assessment of the properties of active motor units [46, 47]. Nevertheless, neither the MFCV raw measure nor its normalized to M‐wave CV correlated with any measure of RTD, as previously reported for knee extensors [23].

Regardless of the method used to infer it, our data (in line with the literature) reinforce the idea that muscle excitation, or more broadly, the net neural drive to motor units, is a crucial factor in rapid torque production during the early phase of a contraction [9].

### 4.4 Mechanical and Structural Contributions

Our data suggest that patellar tendon stiffness plays an important role in determining the explosive strength of the knee extensor muscles, explaining about 25.9%, 33.8%, 21.6%, 16.1%, and 13.3% of the impulse variance at 50, 75, 100, 125, and 150 ms, respectively. Although our data suggested an important role for tendon mechanical properties during the first 100 ms of explosive contractions, it is worth noting that the patellar tendon is a viscoelastic structure that is affected by loading rate. As a consequence, the patellar tendon stiffness calculated during ballistic contractions may differ from that reported in this study, potentially affecting its contribution to determining torque impulse. As contraction time increases and the tendon is fully tensioned, the relative influence of stiffness decreases, and contractile and architectural factors become more prominent [19]. Although some authors have previously emphasized the importance of tendon stiffness in determining explosive strength capacity [17, 19, 48, 49], others have not observed any correlations [18]. We speculate that these discrepancies may be due to differences in the tendon measured (patellar vs. Achilles), in the method for stiffness assessment (torque‐matched, maximal efforts, or explosive contraction), and in the RTD/impulse metrics used.

### 4.5 Muscle Size and Fascicle Behavior

Muscle thickness is a proxy for muscle volume, and the relationship between muscle mass and (late) RTD is already documented [21]. Since generating high torque requires longer periods, our results seem reasonable. In fact, muscle thickness accounted for roughly 0.9% of the impulse variance at 50 ms, 6.5% at 75 ms, 15% at 100 ms, 20.4% at 125 ms, and 24.8% at 150 ms. Previously, Maden‐Wilkinson et al. [21] found that quadriceps muscle size and RTD shared up to ~0.30% of the variance at 100 ms and ~48% at 150 ms. In Coratella et al. [50], the vastus intermedius muscle thickness has predicted ~59% of the variance in RFD during the 100–150 ms epoch. More indirectly, Andersen and Aagaard [12] had reported that maximal voluntary strength (closely related to muscle cross‐sectional area) explained just ~18%–29% of the variance in RFD within the first 50 ms, but its influence grew to ~57% by 100 ms and ~78% by 200 ms. In this sense, our study aligns with the progressive contribution of muscle mass to rapid torque generation across successive time windows.

Regarding muscle behavior, our results show that the fascicle shortening velocity explained impulse variance for ~6.4%, ~13.7%, ~25.5%, ~15.8%, and ~27.8% from 50 to 150 ms. These values, especially from 100 ms onward, are consistent with ranges reported in previous studies that emphasized the contribution of fascicle shortening velocity to RTD (R^2^ = 0.24–0.48) [22]. To note, the *β* coefficients reported in the present study are negative, indicating that lower fascicle shortening velocities are associated with higher impulse production. Since a negative association between fascicle shortening velocity and impulse (or RTD) represents an unusual finding, the following interpretations must be taken with caution.

First, a negative association does not necessarily imply that “slower fibers are better”. According to the F‐V relationship, the force‐velocity potential (i.e., the maximum force a muscle can achieve in a movement) is higher at lower velocities. Therefore, it is possible to assume that the participants operating at lower fascicle shortening speeds were those with the higher F‐V potential. Complementarily, the knee angle used in the present study (85°; 180° = full knee extension) placed the quadriceps in a relatively lengthened configuration compared with more extended setups. This may have increased passive tension and reduced the influence of muscle‐tendon slack on early torque transmission, a mechanism known to affect electromechanical delay and the timing of torque transmission [51, 52]. Therefore, slower fascicle shortening may partly reflect more effective torque transmission under a pre‐tensioned muscle–tendon structure, rather than an intrinsically slower capacity. Finally, the negative association may reflect inter‐individual differences in gearing strategies. On a side note, fascicle shortening velocity in our sample was not significantly correlated with pennation angle, belly gearing, or patellar tendon stiffness, suggesting that it is not a simple surrogate for these other morphological features.

### 4.6 RTD_peak_

Despite the analysis of time‐locked explosive torque being usually preferred because it is more informative [9], RTD_peak_ is the most widely adopted variable in the literature [10, 53]. Since RTD_peak_ occurred on average at 54 ms, we computed two regression models. The one including predictors computed over the 0–75 ms window explained slightly more variance than the model based on the 0–50 ms window (R^2^ = 0.70 vs. 0.61). Based on the 0–75 regression model, RTD_peak_ seems governed primarily by muscle–tendon mechanics with patellar tendon stiffness as the primary contributor (~33% of variance), followed by fascicle velocity (~18%) and muscle thickness (~12%), while only a marginal role for muscle excitation (~8%). The latter appears surprising since the correlation between RTD_peak_ and EMG_vasti_ 0–75 was larger (*r* = 0.53, *r* ^2^ = 0.28) (Table S1). Despite a strong correlation with impulse (*r* = 0.65–0.93), the early occurrence and the use of the same predictors as for impulse regression at 0–75 ms, RTD_peak_ emerges as a distinct outcome measure from time‐matched impulse.

The differences between the RTD_peak_ models with predictors at 0–50 or 0–75 ms are due to two time‐dependent factors (muscle excitation and fascicle shortening velocity), since muscle thickness and tendon stiffness were identical across these models. However, EMG showed very similar standardized coefficients and R^2^ in the two models (Tables S3 and S5), whereas fascicle shortening velocity changed from negligible to moderately associated in the 0–75 ms model, with a corresponding increase in the R^2^. Therefore, the better fit of the 0–75 ms model is more likely to depend on fascicle shortening velocity. Since it was computed as the slope of the shortening‐time curve starting at torque onset, the 0–75 ms window captured fascicle shortening around and partly after the average occurrence of RTD_peak_. Thus, its interpretation needs caution and the higher R^2^ of the 0–75 ms model may reflect a combination of improved EMG timing, broader capture of early fascicle behavior, and sampling/model uncertainty.

### 4.7 Limitations

Due to dropouts and ultrasound data loss (respectively 2 and 6), the subject‐to‐predictor ratio in the linear regression was 6:1, violating the rule of thumb indicating a minimum of 10:1. However, a fundamental research article on linear regression [54] defends the robustness of regressions with a minimum of two subjects per predictor, while advising that adjusted R^2^ be reported (Table S3).

Muscle architecture and behavior were evaluated in the vastus lateralis to represent a proxy of the entire quadriceps behavior. Although this is a strong assumption, it is widely used in the literature since the reliability of ultrasound data collected on vastus medialis, vastus intermedius, and rectus femoris during contractions (especially explosive) is poor and represents a technical challenge. Besides, VL geometrical parameters are typically more correlated with knee extensor torque than those of the other quadriceps muscles [55, 56].

Moreover, because we employed brief, ballistic muscle contractions (< 200 ms), decomposition of HD‐sEMG signals from vastus lateralis into individual motor unit spike trains was not feasible despite the use of a previously validated algorithm. As acknowledged in the discussion, the amount of variance explained by the normalized RMS of EMG (here and in previous studies) may be lower than that explained by studies that used motor unit metrics. In this regard, future studies on the determinants of explosive strength applying multi‐domain approaches should include motor‐unit metrics.

Finally, tendon stiffness was measured during MVT. Although well‐established in the literature, this procedure cannot capture tendon behavior under high loading rates, which occur during explosive contractions, affecting the precision of the final stiffness values [18]. Measuring tendon stiffness during explosive contractions would require high sampling rate ultrasonography [57], which was not available for this study. Nevertheless, our measurements captured inter‐individual differences that were strongly related to impulse (*r* = 0.50–0.77; Table S1), suggesting that tendon stiffness computed as the slope of the torque‐elongation curve may be partially considered a proxy for real tendon behavior in maximal loading rates. Future work should assess tendon stiffness using ultra‐fast ultrasound during rapid contractions [57] to determine more precisely the explosive torque production.

## 5 Conclusions

This study shows that a multi‐domain set of predictors comprising muscle excitation, muscle thickness, fascicle shortening velocity and patellar tendon stiffness helps to elucidate the determinants of rapid knee extension torque production. Although improvements are possible, these findings can be considered as a first attempt at overcoming the fragmentation of data in the literature on the determinants of explosive strength. Specifically, the herein study suggests that early explosive torque output may be significantly determined by tendon stiffness in addition to the well‐established muscle excitation. As the time from contraction onset increases, the role of muscular traits and behavior progressively takes over, while muscle excitation and tendon stiffness lose their centrality.

Altogether, our results remark on the importance of considering force expression as an interplay between different neuromechanical parameters, especially when interpreting analysis coming from one single domain.

## 6 Perspectives

In general, the present findings support the notion that to improve explosive strength, training interventions should increase tendon stiffness [58], early muscle excitation [9, 58], and muscle size [6], possibly through strategies spanning from classic resistance training to explosive types of training.

Notably, this cross‐sectional approach identifies factors associated with between‐subject differences in explosive strength, but it cannot establish whether changes in these factors drive the within‐subject adaptations. In the future, multi‐domain interventions are needed to test whether the determinants identified here still hold as mediators of changes in explosive force production. Furthermore, future studies should address sex‐related differences in explosive force production as predictors may vary across sexes, and a sensitivity analysis suggested this may be the case only at late time windows.

From a statistical standpoint, exercise science research would, in general, benefit from employing dominance analysis to assign relative weights of different predictors of an outcome whether it is related to performance or health. More specifically, the proposed approach may be implemented and refined to better characterize the interplay of mechanisms underlying impairments and the loss of strength (explosive or non‐explosive) in populations with specific conditions or older adults.

## Funding

The authors have nothing to report.

## Ethics Statement

The study was approved by the Ethical Advisory Committee of the University of Turin (approval number 0528472, September 16, 2024) in accordance with the Helsinki Declaration.

## Conflicts of Interest

The authors declare no conflicts of interest. The results of the study are presented clearly, honestly, and without fabrication, falsification, or inappropriate data manipulation. The results of the present study do not constitute endorsement by the American College of Sports Medicine.

## Supporting information


**Figure S1:** sms70328‐sup‐0001‐Supinfo.docx.
**Table S1:** Pearson's correlation coefficients (*r*) between the main metrics and time‐locked or RTD_peak_ impulse.
**Table S2:** Correlation coefficients (*r*) between VL fascicle shortening velocity (V_f_) and other metrics.
**Table S3:** Multiple linear regression standardized beta coefficients (β) at all time windows.
**Table S4:** Commonality coefficients of model R^2^ across time and at RTD_peak_.
**Table S5:** Dominance analysis (LMG) relative importance of determinants across time windows and RTD_peak_ models.

## Data Availability

The data that support the findings of this study are available from the corresponding author upon reasonable request.
