# Supplementary material for: Rapid Torque Production of the Knee Extensors: An Integrative Analysis of Neuromuscular and Muscle–Tendon Determinants
Source: Scand J Med Sci Sports. 2026 Jul 2;36(7):e70328. doi: 10.1111/sms.70328 (PMC13326521; doi:10.1111/sms.70328)
Supplement: Supplementary file 1 — Figure S1: sms70328‐sup‐0001‐Supinfo.docx. Table S1: Pearson's correlation coefficients (r) between the main metrics and time‐locked or RTDpeak impulse. Table S2: Correlation coefficients (r) between VL fascicle shortening velocity (Vf) and other metrics. Table S3: Multiple linear regression standardized beta coefficients (β) at all time windows. Table S4: Commonality coefficients of model R2 across time and at RTDpeak. Table S5: Dominance analysis (LMG) relative importance of determinants across time windows and RTDpeak models. [file SMS-36-e70328-s001.docx]

# Supplementary Material

## Bivariate correlation analysis

**Supplementary Table 1 - Pearson's correlation coefficients (*r*) between the main metrics and time-locked or RTD_peak_ impulse**

| Metric | Impulse50 | Impulse75 | Impulse100 | Impulse125 | Impulse150 | RTD_peak_50 | RTD_peak_75 |
| --- | --- | --- | --- | --- | --- | --- | --- |
| Selected metrics | | | | | | | |
| Th_m_ | 0.07 | 0.33 | **0.47^*^** | **0.54^**^** | **0.58^**^** | **0.44^*^** | **0.44^*^** |
| k_pt_ | **0.60^**^** | **0.77^***^** | **0.58^**^** | **0.55^**^** | **0.50^*^** | **0.75^***^** | **0.75^**^** |
| V_f_ | 0.27 | **-0.52^**^** | **-0.53^**^** | **-0.48^*^** | **-0.59^**^** | -0.10 | **-0.56^*^** |
| EMG_vasti_ | **0.66^***^** | **0.64^***^** | **0.50^**^** | **0.43^*^** | 0.35 | **0.50^**^** | **0.53^*^** |
| Contractility metrics | | | | | | | |
| RTD_peak_ | **0.65^***^** | **0.87^***^** | **0.93^***^** | **0.93^***^** | **0.91^***^** | — | — |
| MVT | 0.20 | **0.51^**^** | **0.68^***^** | **0.78^***^** | **0.85^***^** | **0.64^***^** | **0.64^**^** |
| Octet-evoked RTD50 | 0.16 | **0.48^**^** | **0.65^***^** | **0.75^***^** | **0.81^***^** | **0.69^***^** | **0.69^**^** |
| Octet-evoked RTD_peak_ | 0.21 | **0.46^**^** | **0.60^***^** | **0.67^***^** | **0.70^***^** | **0.69^***^** | **0.69^**^** |
| Unselected metrics | | | | | | | |
| Fascicle length | 0.09 | 0.17 | 0.21 | 0.21 | 0.21 | 0.16 | 0.16 |
| Pennation angle | -0.19 | 0.02 | 0.14 | 0.23 | 0.31 | 0.18 | 0.18 |
| Muscle shortening velocity | 0.31 | **-0.45^*^** | **-0.41^*^** | -0.37 | **-0.55^**^** | -0.07 | -0.45 |
| Muscle belly gearing | **0.49^*^** | **0.47^*^** | **0.51^*^** | **0.52^**^** | **0.41^*^** | **0.50^*^** | **0.66^**^** |
| M-max_VL_ | **0.43^*^** | **0.52^**^** | **0.51^**^** | **0.50^**^** | **0.48^**^** | **0.44^*^** | **0.44^*^** |
| M-max_VM_ | 0.32 | **0.45^**^** | **0.50^**^** | **0.52^**^** | **0.53^**^** | **0.45^*^** | **0.45^*^** |
| MFCV_VL_ | -0.04 | -0.09 | -0.08 | -0.25 | -0.29 | -0.16 | -0.12 |
| MFCV_VM_ | -0.31 | -0.33 | -0.34 | -0.34 | -0.30 | -0.06 | -0.33 |
| MFCV_VL_/CV_M-waveVL_ | 0.07 | -0.06 | -0.16 | -0.19 | -0.24 | -0.23 | -0.22 |
| MFCV_VM_/CV_M-waveVM_ | -0.11 | -0.04 | -0.12 | -0.19 | -0.16 | -0.16 | -0.32 |
| Neural efficacy | 0.20 | **0.62^***^** | - | - | - | 0.12 | 0.43 |
| EMD_VL_ (ms) | 0.25 | 0.02 | -0.14 | -0.24 | -0.31 | -0.12 | -0.12 |
| EMD_VM_ (ms) | 0.20 | -0.07 | -0.27 | **-0.39^*^** | **-0.47^**^** | -0.30 | -0.30 |

**Note:** Metrics (rows) are time-matched (or torque-matched) to the outcome as explained in the article. “Impulse50" refers to impulse computed in the interval 0-50 ms and so on. RTD_peak_50 and RTD_peak_75 columns show the correlation between RTD_peak_ and the time-locked metrics (rows) computed from 0-50 and 0-75 ms, respectively. ^*^ = p < .05; ^**^ = p < .01; ^***^ = p < .001;

**Supplementary Table 2 - Correlation coefficients (*r*) between VL fascicle shortening velocity (V_f_) and other metrics**

| Metric | V_f_50 | V_f_75 | V_f_100 | V_f_125 | V_f_150 |
| --- | --- | --- | --- | --- | --- |
| Fascicle length | 0.18 | 0.076 | 0.093 | 0.119 | 0.102 |
| Pennation angle | -0.393 | 0.020 | -0.059 | -0.334 | -0.123 |
| Muscle thickness | -0.096 | -0.074 | -0.104 | -0.250 | -0.148 |
| EMG_vasti_ | 0.117 | -0.203 | -0.027 | 0.096 | -0.086 |
| k_pt_ from 0-50% MVT | -0.038 | **-0.409^*^** |  |  |  |
| k_pt_ from 50-100% MVT |  |  | 0.008 | -0.242 | -0.131 |
| Muscle shortening velocity | **0.997^***^** | **0.980^***^** | **0.964^***^** | **0.968^***^** | **0.980^***^** |
| Muscle belly gearing | 0.174 | -0.334 | -0.239 | -0.369 | **-0.508^*^** |
| MVT | -0.273 | -0.316 | -0.385 | **-0.405^*^** | -0.393 |
| RTD_peak_ | -0.103 | **-0.561^*^** | **-0.505^*^** | **-0.532^*^** | **-0.571^**^** |
| Octet-evoked RTD50 | -0.228 | -0.370 | -0.401 | **-0.480^*^** | **-0.444^*^** |
| Neural efficacy | -0.002 | -0.269 | -0.192 | -0.077 | -0.197 |
| Octet-evoked RTD_peak_ | -0.196 | -0.331 | -0.310 | -0.387 | -0.365 |

**Note:** Bivariate correlations between vastus lateralis fascicle shortening velocity and architectural, mechanical, and neuromuscular variables (e.g., fascicle length, pennation angle, muscle thickness, patellar tendon stiffness, muscle shortening velocity, belly gearing, MVT, RTD_peak_, evoked RTD_peak_, EMG indices). p < .05; p < .01; p < .001**.**

## Linear regression

**Supplementary Table 3 - Multiple linear regression standardized beta coefficients (β) at all time windows**

| Predictor | impulse50 | impulse75 | impulse100 | implulse125 | impulse150 | RTD_peak_50† | RTD_peak_75 |
| --- | --- | --- | --- | --- | --- | --- | --- |
| Th_m_ | -0.78 | 0.18 | **0.32^*^** | **0.43^*^** | **0.46^**^** | 0.22 | 0.27 |
| k_pt_ | **0.46^*^** | **0.45^**^** | **0.37^*^** | 0.23 | 0.21 | **0.60^**^** | **0.45^*^** |
| V_f_ | 0.23 | -0.24 | **-0.49^***^** | **-0.35^*^** | **-0.48^**^** | -0.08 | **-0.32^*^** |
| EMG_vasti_ | **0.43^*^** | **0.39^**^** | **0.32^*^** | **0.34^*^** | 0.19 | 0.16 | 0.18 |
| Model R^2^ | 0.617^***^ | 0.762^***^ | 0.737^***^ | 0.609^***^ | 0.695^***^ | 0.613^***^ | 0.702^***^ |
| Adjusted R^2^ | 0.537^***^ | 0.712^***^ | 0.682^***^ | 0.526^***^ | 0.631^***^ | 0.532^***^ | 0.640^***^ |

**Note:** The table reports standardized beta coefficients (β) from linear regressions applied to all time-locked impulses each with its predictors set matched per epoch. The table also reports RTD_peak_ linear regression models with predictor sets from 0–50 ms and 0–75 ms. The last row shows the model's explained variance, R².
† **=** The regression model violated the homoscedasticity assumptions; thus, we flag heteroscedasticity-consistent alp-values (HC3) for those models. Standard OLS p-values are denoted for all other models using ^*^ = p < .05; ^**^ = p < .01; ^***^ = p < .001.

## Commonality analysis

**Supplementary Table 4 – Commonality coefficients of model R^2^ across time and at RTD_peak_**

| **Component** | **Impulse50** | **Impulse75** | **Impulse100** | **Impulse125** | **Impulse150** | **RTD_peak_50** | **RTD_peak_75** |
| --- | --- | --- | --- | --- | --- | --- | --- |
| Unique to Th_m_ | 0.005 | 0.026 | 0.075 | 0.135 | 0.161 | 0.039 | 0.058 |
| Unique to k_pt_ | 0.147 | 0.118 | 0.100 | 0.038 | 0.035 | 0.245 | 0.116 |
| Unique to V_f_ | 0.054 | 0.048 | 0.234 | 0.110 | 0.221 | 0.006 | 0.086 |
| Unique to EMG | 0.143 | 0.115 | 0.086 | 0.098 | 0.033 | 0.020 | 0.024 |
| Common to Th_m_, k_pt_ | 0.004 | 0.098 | 0.160 | 0.116 | 0.131 | 0.157 | 0.134 |
| Common to Th_m_, V_f_ | 0.002 | -0.005 | 0.042 | 0.037 | 0.055 | 0.002 | -0.010 |
| Common to k_pt_, V_f_ | -0.010 | 0.088 | -0.022 | 0.029 | 0.010 | 0.006 | 0.113 |
| Common to Th_m_, EMG_vasti_ | 0.019 | -0.022 | -0.038 | -0.052 | -0.028 | -0.010 | -0.016 |
| Common to k_pt_, EMG_vasti_ | 0.246 | 0.179 | 0.100 | 0.056 | 0.022 | 0.152 | 0.089 |
| Common to V_f_, EMG_vasti_ | 0.026 | 0.003 | 0.022 | -0.019 | 0.020 | -0.002 | 0.002 |
| Common to Th_m_, k_pt_, V_f_ | -0.005 | 0.033 | 0.014 | 0.079 | 0.049 | 0.009 | 0.053 |
| Common to Th_m_, k_pt_, EMG_vasti_ | -0.022 | -0.020 | -0.024 | -0.010 | -0.010 | 0.000 | -0.016 |
| Common to Th_m_, V_f_, EMG_vasti_ | 0.007 | 0.003 | -0.014 | -0.002 | -0.013 | 0.000 | 0.001 |
| Common to k_pt_, V_f_, EMG_vasti_ | 0.009 | 0.106 | -0.002 | 0.006 | 0.011 | -0.006 | 0.080 |
| Common to Th_m_, k_pt_, V_f_, EMG_vasti_ | -0.007 | -0.008 | 0.005 | -0.012 | -0.001 | -0.004 | -0.009 |
| **Model R^2^** | **0.617^***^** | **0.762^***^** | **0.737^***^** | **0.609^***^** | **0.695^***^** | **0.613^***^** | **0.702^***^** |

**Note**: Commonality analysis coefficients decompose each model R² into unique and shared variance components for the predictors (Th_m_, k_pt_, V_f_, and EMG_vasti_) across all time windows and RTD_peak_ models. The color scale from blue to red indicates, respectively, the greater positive and negative contributions to the total variance explained by the model (R^2^, in the last row). ^*^ = p < .05; ^**^ = p < .01; ^***^ = p < .001.

# Dominance analysis

**Supplementary Table 5 - Dominance analysis (LMG) relative importance of determinants across time windows and RTD_peak_ models**

| Predictor | Impulse50 | Impulse75 | Impulse100 | Impulse125 | Impulse150 | RTD_peak_50 | RTD_peak_75 |
| --- | --- | --- | --- | --- | --- | --- | --- |
| Th_m_ | 0.009 | 0.065 | 0.15 | 0.204 | 0.248 | 0.115 | 0.121 |
| k_pt_ | 0.259 | 0.338 | 0.216 | 0.161 | 0.133 | 0.402 | 0.32 |
| V_f_ | 0.064 | 0.137 | 0.255 | 0.158 | 0.278 | 0.009 | 0.18 |
| EMG_vasti_ | 0.285 | 0.223 | 0.115 | 0.086 | 0.036 | 0.087 | 0.081 |
| Model R^2^ | 0.617^***^ | 0.762^***^ | 0.737^***^ | 0.609^***^ | 0.695^***^ | 0.613^***^ | 0.702^***^ |

**Note:** the table reports the relative importance (LMG) of Th_m_, kpt, V_f_, and EMG_vasti_ across time windows and RTD_peak_ models. Values represent each predictor’s share of explained variance, alongside total model R².

**Supplementary Figure 1**


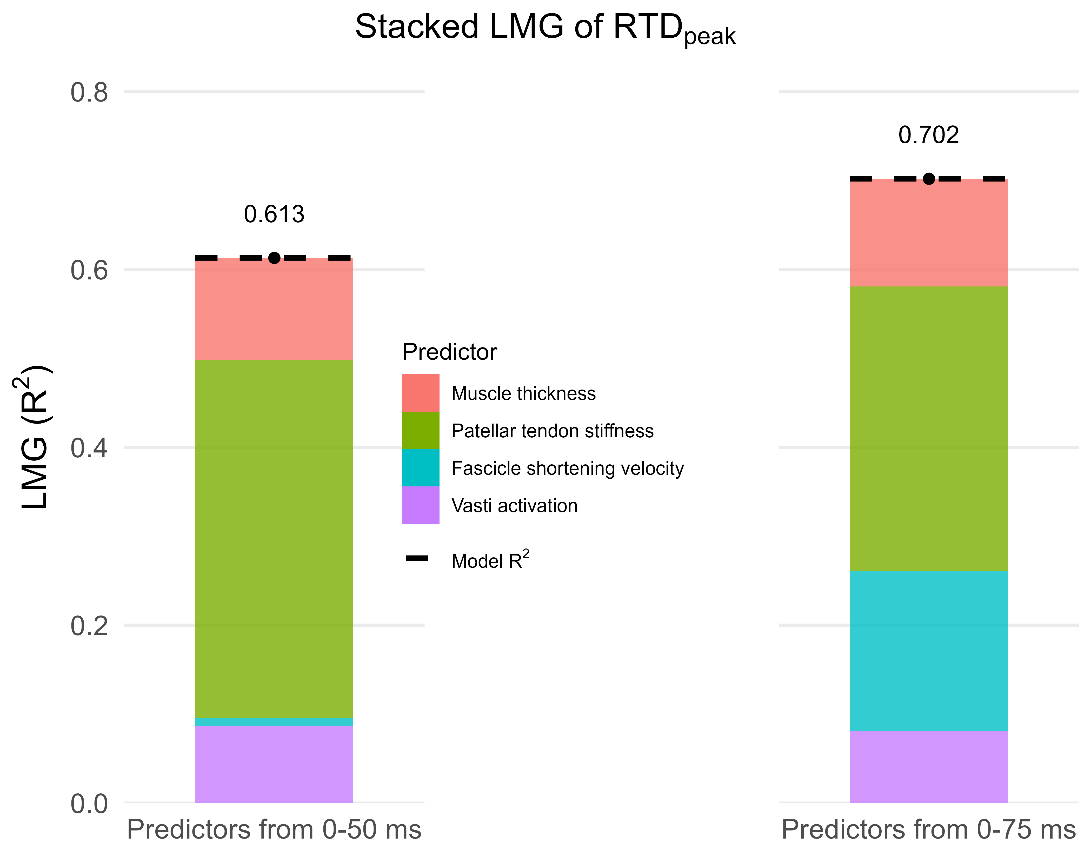


**Note: Stacked-bar visualization of predictor contributions to explained variance (model R²)** for the impulse0–50 ms model (left bar; total ≈ 0.61) and the RTD_peak_ model using the 0–75 ms predictor set (right bar; total ≈ 0.70), with coloured segments showing Th_m_, k_PT_, V_f_, and EMG_vasti_ contributions.
